# Vertebrate food products as a potential source of prion-like α-synuclein

**DOI:** 10.1038/s41531-017-0035-z

**Published:** 2017-11-24

**Authors:** Bryan Andrew Killinger, Viviane Labrie

**Affiliations:** 1 Center for Neurodegenerative Science, Van Andel Research Institute, Grand Rapids, MI USA; 2 Centre for Addiction and Mental Health, Toronto, ON Canada

## Abstract

The aberrant aggregation of the protein α-synuclein is thought to be involved in Parkinson’s disease (PD). However, the factors that lead to initiation and propagation of α-synuclein aggregation are not clearly understood. Recently, the hypothesis that α-synuclein aggregation spreads via a prion-like mechanism originating in the gut has gained much scientific attention. If α-synuclein spreads via a prion-like mechanism, then an important question becomes, what are the origins of this prion-like species? Here we review the possibility that α-synuclein aggregation could be seeded via the ingestion of a prion-like α-synuclein species contained within food products originating from vertebrates. To do this, we highlight current evidence for the gut-to-brain hypothesis of PD, and put this in context of available routes of α-synuclein prion infectivity via the gastrointestinal (GI) tract. We then discuss meat as a ready exogenous source of α-synuclein and how certain risk factors, including inflammation, may allow for dietary α-synuclein to pass from the GI lumen into the host to induce pathology. Lastly, we review epidemiological evidence that dietary factors may be involved in PD. Overall, research to date has yet to directly test the contribution of dietary α-synuclein to the mechanism of initiation and progression of the disease. However, numerous experimental findings, including the potent seeding and spreading behavior of α-synuclein fibrils, seem to support, at least in part, the feasibility of an infection with a prion α-synuclein particle via the GI tract. Further studies are required to determine whether dietary α-synuclein contributes to seeding pathology in the gut.

## Introduction

### Prion-like aggregation of α-synuclein in Parkinson’s disease (PD): from gut to brain

PD is a progressive neurodegenerative disease affecting millions of people worldwide. PD symptoms include disruptions of motor behavior such as postural bradykinesia, rigidity, and resting tremors,^1^ as well as non-motor symptoms, including constipation, sleep disturbances, depression, anxiety, and impaired olfaction.^2^ Motor symptoms result from the significant loss of dopamine neurons in the substantia nigra (SN).^3,4^ The exact molecular causes of dopamine neuron loss in the SN remains unclear, but the aberrant aggregation of the protein α-synuclein is thought to be involved.^5,6^ The hallmark of PD is α-synuclein-containing intracellular inclusions found both in neuronal cell bodies and neuronal processes of the brain, termed “Lewy pathology,” collectively.

α-Synuclein is a small aggregation prone protein of unknown biological function.^7–9^ In vivo, α-synuclein forms several small homo-oligomers (i.e., dimer, tetramer, octamer) that are likely important for its endogenous functions, such as intracellular vesicular trafficking.^10,11^ α-Synuclein can also form progressively massive insoluble amyloid fibrils in vitro that consist of hundreds of α-synuclein proteins in a stable β-sheet conformation.^12^ Several species of α-synuclein including the monomer, dimer, tetramer, protofibrils, and full length fibrils have been implicated in PD.^13–17^ The shift from endogenous α-synuclein monomer to oligomeric and fibril states have been shown to be particularly neurotoxic,^18^ though further research is required to determine what multimeric species or conformer of α-synuclein, if any, are genuinely disease causing.

There is evidence that fibrils are a disease causing species of α-synuclein.^18,19^ Synthetic fibrils, referred to as preformed fibrils, can be transmitted from cell-to-cell, and when injected into the brain can recapitulate symptoms and pathology of PD.^18,19^ The spread of α-synuclein fibrils likely involves a templating mechanism; where either intact, or fragmented (i.e., protofibrils), fibrils leave a donor cell, are subsequently taken up by a host cell, and seed further aggregation via interaction with the endogenously expressed α-synuclein of the host cell.^20–25^ The accumulation of intracellular fibrils may be inherently toxic, or alternatively, fibrils may exert toxicity by indirect mechanisms, such as inhibiting the normative function of endogenous α-synuclein.^26^ Disease subtypes and progression may depend on the specific “strain” or conformation of the fibril species, which affects α-synuclein structure, level of toxicity, in vivo propagation, and neuropathology.^27^ The cell-to-cell spread of fibrillary α-synuclein offers an explanation for the observations that α-synuclein pathology is observed in healthy neurons grafted into the striatum of PD patients.^28–30^ Indeed, α-synuclein has been shown to be released from neurons,^19,31–34^ followed by subsequent spread to neurons within close proximity.^35,36^ The spreading behavior of α-synuclein pathology has led many researchers to hypothesize that PD results from a “prion-like” mechanism.^37–41^ However, a “prion-like” mechanism of PD is somewhat contradicted by the observation in some PD patients whose pathology does not adhere to the staging scheme proposed by Braak.^42–45^ Together, the prion-hypothesis of PD seems mechanistically viable and deserving of systematic study.

Prions are transmittable infectious particles comprised solely of protein (i.e., devoid of nucleic acids), namely prion protein (PrP), that cause fatal diseases.^46^ In general, the term “prion” refers to infectious proteins which propagate a disease by causing protein conformational changes. For PrP to become a disease causing protein it must first convert from α-helical structure to a stable β-sheet conformation.^47–49^ This disease causing β-sheet isoform of PrP is referred to in the literature as PrP^Sc^. Once an animal is inoculated with PrP^Sc^ it can spread from cell-to-cell both within and between species.^50^ Environmental prions are infectious and can cause prion disease, however, prion disease can also be inherited.^51^ For example, prion diseases like Creutzfeldt–Jakob disease (CJD) and variant CJD (vCJD) occur both through a familial pattern of inheritance and sporadically.^52^ Although considerable heterogeneity exists between the pathology of prion diseases,^53^ generally PrP^Sc^ causes extensive damage to the nervous system, including vacuolation (resulting in the spongiform appearance of brain tissue), astrocytic gliosis, and PrP deposition, which together ultimately lead to death.^54^

In the context of α-synuclein, the term “prion-like” refers to the capacity of misfolded alpha-synuclein to transfer from cell-to-cell within an organism and act as template for further aggregation. Currently, α-synuclein and aggregates of α-synuclein are not considered as infectious, communicable agents, and as a result it is debated whether these are true prions. Indeed, exposure to human growth hormone potentially contaminated with α-synuclein aggregates was not associated with PD.^55^ However, there are many similarities between the molecular behavior of α-synuclein and that of the classic prion, PrP^Sc^.^9,41,56,57^ First, inoculation of rodents and monkeys with the aggregated α-synuclein or brain lysates from PD patients is sufficient to induce PD-like symptoms, similar to what was observed during seminal studies with PrP^Sc^.^46,58^ Second, α-synuclein fibrils can serve as templates for aggregates,^24,59^ whose structures vary, depending on the sequence of α-synuclein used to produce the template fibril.^60^ Third, the molecular events that lead to the initiation of known prion species involves the loss of α-helical tertiary structure in favor of stable β-sheet conformation, which is a characteristic shared by α-synuclein.^12^ Specifically, in the cell, α-synuclein likely exist in dynamic equilibrium between slightly compact disordered monomer and phospholipid bound form containing an n-terminus α-helical structure. The highly conserved non-amyloid-β component (NAC) domain of α-synuclein is alone sufficient to form pathological fibrils^24,61^ and has been shown to be shielded by the C-terminus region to prevent spontaneous aggregation.^62^ However, circumstances that expose the NAC domain have been found to promote fibril formation, leading to cytotoxicity and pathology.^61,63^ Whatever the pathogenic species of α-synuclein may be, it is clear that misfolding and aggregation, followed by spread in a prion-like manner, is in some way involved in the development of PD.

### Gut as initiation site for α-synuclein pathology

The gut has been hypothesized as an initiating site for α-synuclein pathology.^64^ Originally, Braak et al. proposed that exposure to an unknown pathogen (virus, bacteria, prion, etc.) through the gut epithelial lining could trigger α-synuclein aggregation in enteric neurons, and α-synuclein aggregates could subsequently spread to the brain.^64–68^ This hypothesis was based on the observation that in many, but not all,^69,70^ PD patients Lewy body pathology starts in the dorsal motor nucleus and then progressively spreads throughout the brain.^43,67^ The dorsal motor nucleus is directly connected to the gut via vagal nerve afferents/efferents and therefore, represents a direct physical connection between gut neurons and the central nervous system (CNS). Because the gut is lined with a semi-permeable mucosa, the gut becomes a possible site for the introduction of an unknown pathogen and the subsequent initiation of PD pathology.

Several recent studies have, in part, supported this “gut to brain” hypothesis of PD.^71,72^ α-Synuclein is endogenously expressed in enteric neurons throughout the gastrointestinal (GI) system and PD-like α-synuclein pathology has been documented in intestinal tissues of PD patients^68,73,74^ and prodromal-PD patients.^75^ Local injections of α-synuclein into the vagal nerve of rats can induced PD-like pathology throughout CNS structures.^76^ Similar results have been seen with local injection of α-synuclein into the rodent olfactory bulb,^77^ skeletal muscle,^78^ and intestinal wall.^79^ Bidirectional transport of α-synuclein pathology is likely, because rodent models overexpressing α-synuclein in the midbrain show α-synuclein pathology in the GI system.^80^ PD pathology in the CNS can be induced in rats via GI administration of the neurotoxin rotenone.^81^ The spread of administered or rotenone-induced alpha-synuclein fibrils from the gut to the brain of rodents can be abolished by severing the vagal nerve.^81,82^ The transport of pathological α-synuclein from the gut to the brain via axonal transport has also been directly observed in rats by utilizing live cell imaging.^79^ In concordance with the apparent spread of α-synuclein pathology via the vagal nerve, some epidemiological studies found that a full truncal, but not super-selective, vagotomy was associated with a decreased incidence of PD in humans.^83,84^ Furthermore, pathology has been detected in the GI tract 8 years before the clinical onset of PD^85^ (though this finding has not yet been replicated). Despite these findings, the hypothesis that pathology begins in the gut of human patients and then spreads to the CNS has not been documented unequivocally. Several reports have documented α-synuclein pathology in the GI tract of healthy individuals, bringing into question whether peripheral pathology is related to PD.^86,87^ Furthermore, the detection of α-synuclein pathology biomarkers in peripheral biopsies of GI tissue have so far proven unreliable.^87^ However, a recent report determined that α-synuclein pathology in colonic biopsies can accurately distinguish between PD from control patients provided neuronal elements were represented in the sample and the pathologist is adequately trained.^88^ Therefore, though evidence does suggest that peripheral pathology/insult may lead to CNS pathology, further work is required to clarify to the role of the GI tract in PD.

### Routes of prion infectivity in the gut

How might α-synuclein prion particles from food infect a human host? One possibility is transcellular antigen sampling via microfold cells (M cells), dendritic cells, and to some extent columnar enterocytes.^89^ Antigen sampling is the process of controlled absorption of luminal contents and the subsequent transport of these particles to the gut-associated lymphoid tissue (GALT) to stimulate B-cell IgA production.^89^ Antigen sampling is crucial for intestinal homeostasis and the distinction between harmful and harmless luminal contents. M cells are specialized immune cells of lymphoid-associated mucosal tissue, and are known to take up concentrated samples of prions.^90,91^ M cell depletion in the gut can abolish PrP^Sc^ uptake by the host.^92^ Cross-species infection of prions occurs more readily in peripheral lymphoid tissue compared to nervous tissue of the CNS.^93^ Lymphoid tissue and associated M cells are found in numerous portions of the GI tract, including in the tonsils, ileum and appendix, potentially providing an entry route for prion uptake. Insoluble multimeric particulates that readily adhere to mucosal surfaces are most effectively taken up by M-cells, a phenomenon that has been exploited for the delivery of oral vaccines.^94,95^ M cells can actively transport particles up to 1 µm, more than sufficient for the uptake of short α-synuclein fibrils (~10–1000 nm).^96^

Dendritic cells, which are primarily antigen presenting cells, can also actively sample GI luminal contents/pathogens.^97^ Dendritic cells migrate between tight junctions and sample the luminal contents directly, including PrP^Sc^.^98^ Sampled luminal antigens are then presented to T cells of the GALT. α-Synuclein binds to lymphocyte-activated activation gene 3 (LAG3) with nanomolar affinity, and binding to LAG3 appears to drive neuropathology.^99^ LAG3 is expressed in T cells,^100^ B cells,^101^ some dendritic cells,^102^ and neurons.^32^ Therefore, misfolded α-synuclein sampled from the intestinal lumen would be sampled and transported to the lymphoid tissue containing LAG3 positive T cells. Subsequent neuronal interaction with T cells could initiate and drive aggregation in the GI tissue.^99^

Leaky gut could be another gateway for the entry of exogenous prion-like particles, including α-synuclein.^103^ Normally the semipermeable barrier throughout the GI tract prevents unwanted exposure to luminal contents. However, under conditions of infection, inflammation, and GI disease the gut barrier can become permeable allowing potentially toxic luminal contents to pass the protective epithelial barrier.^89^ Dietary fiber deprivation has also been shown to degrade the intestinal barrier and enhance pathogen entry.^104^ Hence, a leaky gut could potentially expose submucosal neurites and/or the underlying immune tissues to luminal contents. Recently, it was found that many PD patients contain activated T cells against the n-terminal of α-synuclein, suggesting PD may be a result of autoimmunity against this antigen.^105^ The entry of protein particles through leaky gut has been hypothesized as a factor leading to autoimmunity.^106^ Hence, it is an intriguing possibility that under conditions of a “leaky gut” dietary α-synuclein could enter host and result in an adaptive immune response.

Once in the gut α-synuclein may infect host neurons or immune cells through several mechanisms.^107^ Passive and/or receptor mediated endocytosis could be involved with the uptake of α-synuclein into the host neurons in the gut. Once α-synuclein binds to either LAG3 receptors on neurons^32^ or to the membrane via electrostatic interactions it can be endocytosed into the host enteric neurons. LAG3 receptors have been shown to preferentially mediate the uptake of fibrillary forms of α-synuclein.^32^ From the early endosome α-synuclein is then trafficked to the lysosome. How α-synuclein might escape lysosomal degradation is unknown, but it could be through the ability of α-synuclein fibrils to disrupt lipid bilayer structures.^108^

α-Synuclein fibrils could also be taken up by cells in the gut via micropinocytosis.^109^ Cellular uptake of exogenous α-synuclein fibrils by micropinocytosis has been shown to be mediated by cell surface heparin sulfate proteoglycans^109^ which are ubiquitously expressed in most cell-types throughout the body. Heparin sulfate proteoglycans are crucial for maintaining the intestinal epithelial barrier.^110,111^ Cell surface proteoglycans can mediate the uptake of unwanted bacterial and virus through the digestive tract.^112^ PrP^sc^ binds to cell surface proteoglycans which is thought to drive propagation.^113,114^ Therefore, alpha-synuclein could be taken up by the host via interactions with cell surface molecules such as heparin sulfate proteoglycans.

### Dietary sources of α-synuclein

Nearly all of the vertebrate species we eat including Bos taurus (cow), Gallus gallus (chicken), Sus scrofa (pig), and a variety of fish species express α-synuclein.^115,116^ α-Synuclein protein is highly conserved with 97.9%, 94.3%, and 86.7% sequence homology to the human protein for pig, cow, and chicken, respectively. α-Synuclein is most abundantly expressed in the brain, but is also expressed in many tissues throughout the body with the exception of the liver.^7,117–119^ Food products containing α-synuclein are most commonly meat products comprised of skeletal muscle.^120^ Dairy products such as milk may contain trace amounts of α-synuclein, although the unambiguous identification of α-synuclein in the milk proteome is lacking.^121^ Several less commonly consumed food products such as calves’ brain (i.e., sweet breads) and bone marrow from cows are a rich source of α-synuclein because of the abundant α-synuclein expression in neurons^122,123^ and hemopoietic cells/megakaryocytes,^124^ respectively.

Skeletal muscle of the cow is a source of α-synuclein commonly found in the human diet.^120^ In such tissue, α-synuclein is most abundantly expressed in neurons, but is also expressed in lower abundance in other cell types, including hemopoietic cells and myocytes.^120^ The abundant expression of α-synuclein in hemopoietic cells likely accounts for the observed abundance of α-synuclein in various blood cells^125^ (erythrocytes and platelets). Neurons, blood cells, and myocytes are all found in the skeletal muscle tissues eaten by humans. Therefore, several cell types found within muscle contain α-synuclein and contribute to the total amount of ingested α-synuclein. Peripheral neurons abundantly express α-synuclein^126^ and therefore peripheral motor axon projections to myocytes^119^ also contribute to α-synuclein in meat. Erythrocytes are another potential source of α-synuclein^125^ in meat products as an estimated 2–9 mL blood/kg meat remains following slaughter.^127^ The presence of alpha-synuclein in the animal food products consumed by humans begs the question: is a potential source for prion-like α-synuclein from the food that we eat?

### Vertebrate food products may contain disease-associated α-synuclein

**Fig. 1:**
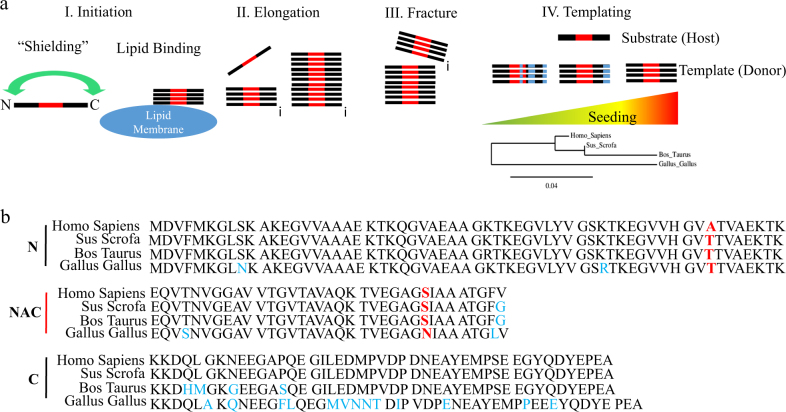
Potential templating mechanism of α-synuclein from vertebrate meat products. **a** The pathogenic aggregation of α-synuclein may involve several steps. (I) The mechanism for pathogenic α-synuclein aggregation is unknown. However, several cellular factors such as transient interactions between the N-terminus and C-terminus (“Shielding”) or lipid binding may play a role in the initial aggregation; inhibitory or promoting. Also, interactions between non-amyloid component (NAC) domains is necessary and sufficient for aggregation (Colored red). (II) Once a self-propagating oligomer (depicted here as a protofibril) is formed it then elongates by the addition of α-synuclein monomers to the ends of fibril. (III) Mechanical fracturing of the fibril into smaller templates allows for further seeding. (IV) The seeding capacity during the templating step is highly dependent on factors such as homology between the template protein and the substrate protein. Homology of α-synuclein protein is high between human and several animals species humans eat. Phylogenic tree showing α-synuclein protein sequence homology between human (Homo Sapiens), pig (Sus Scrofa), cow (Bos Taurus), and chicken (Gallus Gallus). Reference bar 4% divergence in sequence homology. **b** Specific amino acid sequences for α-synuclein of all species compared. Red denotes variable positions adjacent to the NAC domain that greatly influence cross-species seeding. Blue denotes variable amino acid positions compared to human α-synuclein

All α-synuclein in dietary meat products contains threonine at the amino acid 53 position, while human α-synuclein has alanine at this position. The A53T mutation of human α-synuclein was the first identified to be associated with PD.^128,129^ Patients who carry the A53T mutation develop early onset PD. A53T α-synuclein shows impaired lipid binding and enhanced aggregation properties.^130–134^ The threonine at amino acid 53 of α-synuclein is conserved across most vertebrate species (except several primates), including those animals that are commonly consumed in the human diet^135^ (Fig. 1). Cows, chickens, and pigs all have threonine at the 53 position of α-synuclein.^135^ Because A53T human α-synuclein increases aggregation^134^ and is disease causing in humans,^136^ the ingestion of a close homolog through diet could increase the likelihood of seeding pathology in the gut (described further in Fig. 1). In support of this idea, α-synuclein from vertebrate species other than human are more prone to rapid fibrillization.^137^ Alternatively, α-synuclein from other species may be particularly poor, even inhibitory, for the formation of aggregates.^137,138^ Recent findings utilizing several chimeric α-synuclein proteins suggest an even more complex relationship; where single amino acid sequence differences between α-synuclein fibril seeds and α-synuclein expressed by the host species determine resulting pathology.^139–141^ Surprisingly, sequence homology between the seed and substrate do not exclusively correspond with pathogenicity^139^ suggesting pathogenic seeds can cross species. Together, divergence of α-synuclein at amino acid 53 in vertebrate species may have relevance for subsequent cross-species aggregation seeding.

Oxidation in meat products could further promote the formation of prion α-synuclein particles. Numerous oxidative biological products are found in high abundance in PD brains,^142–144^ suggesting their involvement in PD. Oxidized proteins often form intra/intermolecular covalent bonds with other molecules, including other proteins. α-Synuclein, specifically, can be modified via oxidation to form covalently linked cytotoxic oligomers.^145–148^ Specifically, dityrosine covalent linkages between α-synuclein molecules form low weight oligomers/protofibrils that have been shown to serve as a template to seed α-synuclein aggregation.^145,146,149^ In meat products, proteins become extensively oxidized in a complex process, dependent both on time and environmental catalysts (i.e., exposure to oxygen, enzymes, etc.).^150^ Oxidative products from meat products have been implicated in several human age-dependent diseases, including atherosclerosis^151^ and several cancers.^151–155^ Oxidation of meat proteins can result in extensive protein-protein crosslinking which, in turn, can alter conformation and function of those proteins.^152^ Factors such as aging, grinding, and cooking meat can affect overall oxidation of meat proteins.^156–158^ If α-synuclein in meat products were to become heavily oxidized, this could result in increased protease resistance,^159^ stabilization of β-sheet structure,^47^ and possible oligomer formation.^145^ However an important question remains as to whether any of these oxidized α-synuclein species from meat products could act as a prion. Furthermore, if they can act as a prion, do dietary sources contain a relevant concentration to be pathogenic?^160^

### The species barrier

The zoonotic transfer of prions is considered rare because of the “species barrier”.^161,162^ A major factor influencing the species barrier is the degree of sequence homology between the PrP and the host species.^50,163,164^ Greater sequence homology between the donor and host species PrP increases the likelihood of infectivity.^50^ There is a high degree of sequence homology between human and bovine for PrP (86%^165^), as well as for α-synuclein (94.3%; Fig. 1). It is now clear that PrP^Sc^ can be transmitted between species, including from bovine to humans, most likely via consumption of contaminated meat.^166,167^ It is possible for α-synuclein fibrils to seed pathology across species, although the species barrier does limit this process.^139^ Similar to PrP^sc^, sequence homology between α-synuclein seed and the host protein (i.e., substrate) is proportional to the seeding initiation rate.^139,168^ For example, fibrils grown from full-length human α-synuclein potently seed aggregation of monomeric human α-synuclein, while being relatively ineffective at seeding the aggregation of monomeric mouse α-synuclein.^139^ In vivo there is good evidence that mouse α-synuclein actually inhibits the pathogenic fibrillization of human α-synuclein.^169^ Therefore, a crucial question arises whether a zoonotic α-synuclein fibril conformer exists, and from what conditions it can be generated. Indeed, several fibrils raised from chimeric mutant α-synuclein proteins have been found to potently seed pathology.^139^ Two residues of α-synuclein, namely amino acids 53 and 87, seem to be critical for cross-species seeding of pathology.^139^ A single substitution at either of these positions (i.e., to greater resemble the host protein) restores templating characteristics of the fibril in the host species. Correspondingly, pathology is only observed in rodents overexpressing A53T, and not full length, human α-synuclein.^170^ It is an interesting idea that humans carrying the A53T mutation may be susceptible to templating from exogenous α-synuclein of other species. However, sequence homology is likely not the only factor driving α-synuclein pathology, because then pathology would be expected to spontaneously arise within any organism expressing α-synuclein. For example, α-synuclein concentration in human saliva has been measured as ~100 pg/mL^171^ and humans can produce ~500 mL of saliva everyday,^172^ and therefore, we readily ingest ~50 ng of our own α-synuclein each day. Certainly there are unknown cellular factors besides sequence homology that are required for initiation and formation of pathogenic seeds.^139^

### Epidemiological evidence for a dietary role in PD

Prion disease involving PrP^Sc^, such as CJD or vCJD, can progress rapidly following the onset of initial symptoms, resulting in death within months. In contrast, PD typically progresses slowly over a period of many years, and therefore, the prion properties of α-synuclein are “slow” when compared with PrP^Sc^. The difference in the rates of prion spread makes it difficult to test the hypothesis of a “slow” prion-like α-synuclein, which may not produce clinical symptoms for many years following exposure. Even a “fast” prion like PrP^Sc^ doesn’t always cause disease immediately and can remain dormant for prolonged incubation periods, sometimes exceeding as much as 50 years.^173^ Patients that received contaminated dura mater tissue grafts may not show symptoms anywhere from 1–30 years.^174,175^ This prolonged incubation makes tracking all prion infections exceedingly difficult. Even if the α-synuclein prion was known, and a patient had been exposed to this prion, it would be difficult to predict the progression and age of onset of the disease.

Despite the challenges of detecting causal prion exposures and a limited understanding of the cross-species capacity of animal α-synuclein, there have been some dietary studies linking intake of animal products with PD risk.^176^ Heterocyclic amines are toxic compounds found in cooked meat, and there is some evidence that these compounds are elevated in the brains of PD patients.^177^ Intake of animal fats in adulthood (i.e., 20–30 years prior to onset of the disease) has been associated with a higher PD risk.^178^ Some studies found that animal fat consumption strongly increased PD risk (2–9-fold higher PD risk), although this remains controversial.^179–181^ Furthermore, consumption of dairy products has been found to increase the risk of PD, particularly for men.^182^ A recent study found that in a middle-aged Hawaiian population, milk consumption significantly increased the risk of PD.^183^ A meta-analysis confirmed a positive correlation between milk intake and risk of PD.^184,185^ However, several separate studies failed to show a relationship between milk consumption and PD.^186^ Dietary habits vary geographically, but the incidence of PD is relatively constant when controlling for age,^187,188^ which suggests, in part, that the disease is non-infectious. Furthermore, there have been documented cases of CJD involving PrP^sc^ in northern India, which is a primarily vegetarian culture,^189^ and in patients who had limited contact with meat products.^190^ Therefore, investigation of vegetarian cultures may not be a perfect test of dietary origins of prion-like α-synuclein. Regardless, large-scale epidemiological studies and meta-analyses of dietary contributions to PD may help resolve the controversies related to dietary risk factors for PD.

Causal genes have been identified for PD,^191–193^ but approximately 70% of PD cases are sporadic,^193^ and therefore have no clear monogenic cause. However, evidence for oligogenic contributions to PD risk have been determined.^194^ Furthermore, a combination of risk factors such as sex, age, genetics, and anosmia, when considered together, have a high predictive value for PD.^195^ Together these results seem to suggest that environmental factors contribute little to PD. Alternatively, these risk factors could mediate susceptibility to environmental factors, such as increasing the exposure duration to prion particles. For example, genetic risk factors for PD particularly affect genes involved in the autophagy and lysosomal pathway, which is responsible for protein clearance and degradation.^194,196,197^ α-Synuclein aggregates are cleared by lysosomal degradation,^198^ suggesting that PD pathogenesis involves an aberrant accumulation of misfolded α-synuclein. Similarly, evidence suggests that infection and spread of PrP^sc^ may be augmented by dysfunctional lysosomes.^199,200^ Presumably genetic factors that impair the clearance of exogenous α-synuclein particles that are entering through the gut and that are capable of seeding aggregated forms will consequently increase the likelihood of subsequent pathology. Therefore, genetics, age, and other PD risk factors could “prime” certain individuals to be more receptive to environmentally-derived α-synuclein pathology. Autoimmune disease and viral infections also show oligogenic predispositions in the immune system that control the host’s response to “invading” pathogens.^201,202^ Analogously, genetic predisposition to impaired lysosomal clearance of protein buildup may favor the accumulation of an exogenous α-synuclein prion-like protein.

### Possible risk factors

Many people regularly consume meat and dairy products, but only a small fraction of the general population will develop PD. Therefore, it is unlikely that eating meat products is an independent cause of PD. The accompaniment of certain risk factors, such as inflammation, aging, genetic and epigenetic factors, may provide an opportunity for unwanted dietary α-synuclein to enter the host, and initiate disease.

Much research has shown that the immune system and inflammation are likely involved in PD.^105,203–207^ Systemic inflammation is known to enhance infectivity of PrP^Sc^, and subsequently, inflammation may also trigger and/or accelerate the infection and spread of prion α-synuclein.^208,209^ Specifically, gut inflammation may allow toxic luminal contents to diffuse across intestinal epithelial tight junctions,^210^ increase aggregation,^209^ and promote the spread of aggregates in the gut.^209^

An imbalance in gut microbiome may be involved in PD.^71,211,212^ Fecal transplants from PD patients enhance the α-synuclein pathology and motor dysfunction seen in A53T mutant mice.^71^ The effect of the microbiome on PD pathology and neurological health/function has been shown to involve alterations in the intestinal barrier and activation of immune cells, including microglia.^71,211,212^ Numerous other neurological disorders and neurodegenerative disorders have been linked to imbalances in the microbiome.^213^ Alterations in short-chain fatty acids produced by specific gut microbes can have pro-inflammatory properties in the gut, reduce gastric motility, and increase permeability through intestinal epithelial cell tight junctions;^71,214^ effects that could promote entry of dietary α-synuclein particles from the lumen.

In addition, the gut microbiota may be an exogenous source for prion-like particles to trigger α-synuclein aggregation.^72,215^ Several prion-like peptides expressed by gut microbiota have been identified and found to enhance α-synuclein aggregation in enteric neurons.^72^ However, it still remains unclear if the bacterial peptides are produced in sufficient quantities and the microbiome can induce PD in human beings.

Aging is the most important risk factor for PD, and may increase the likelihood of invasion of harmful antigens in the GI tract.^216^ Age-related GI changes such as slowed gastric emptying, decreased GI tract motility, reductions in protective GI mucous, reductions in luminal digestive enzymes would increase the time of exposure to dietary α-synuclein.^216^ The intestinal epithelial barrier may also become progressively more permeable with age.^217^

Epigenetic regulation of gene structure/function may play a role in the PD.^218–221^ Specifically, dysregulation of PD genes may be controlled by epigenetic mechanisms.^222,223^ In contrast to somatic mutations and copy number variations,^192,224–226^ the epigenome is partially dynamic such that it is modifiable by environmental factors and with aging.^227^ Age-dependent epigenetic alterations have been shown accumulate more rapidly in PD patients.^228^ Furthermore, several PD-associated genes, including parkin (*PARK2*)^226,229^ and α-synuclein^225,230,231^ exhibit dysregulated activity in PD. Transcript expression of both of these PD genes have been shown to be regulated by the epigenetic mark DNA methylation.^220,232–234^ PD risk factors, involving diet,^235^ inflammation^236^ and the microbiome^237^ are known to cause epigenetic changes,^238,239^ and may affect enteric neuron susceptibility to α-synuclein pathology. In addition, the upregulation of α-synuclein expression in enteric neurons by viral infections^240,241^ may also involve epigenetic alterations. Consequently, epigenetic misregulation of PD risk genes may increase the likelihood that dietary α-synuclein sources could seed pathological α-synuclein aggregation in the GI tract.

## Conclusion

**Fig. 2:**
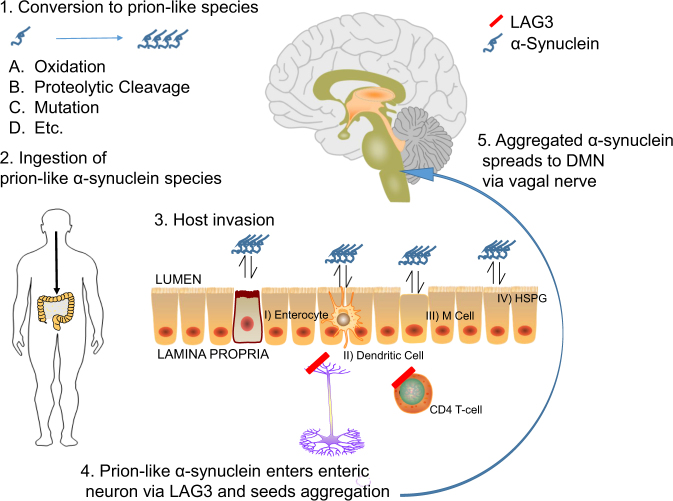
Theoretical schema demonstrating the entry of dietary, prion-like α-synuclein into the gut and subsequent induction of pathology in the brain. Depicted is a stepwise summary of a possible mechanism whereby a disease causing α-synuclein particle may be derived from food products and infect the host. (1) α-Synuclein contained in meat products may undergo several chemical/structural modifications that result in the generation of a pathogenic species. (2) This pathogenic, dietary-derived α-synuclein is then consumed, and (3) infects the host possibly via transcellular uptake through (I) enterocytes, (II) dendritic cells, (III) M cells, or (IV) interactions with heparin sulfate proteoglycans (HSPG). Factors such as inflammation, genetics, and aging may modify the pathogenicity of dietary-related α-synuclein. (4) Once in the digestive tissue these exogenous α-synuclein particles may enter neurons via interactions with the LAG3 receptor. (5) Once in the neurons of the host, dietary-derived α-synuclein may then initiate pathology (i.e., the aggregation of endogenous α-synuclein) which then spreads to the brain via the vagal nerve. LAG3 lymphocyte-activated gene 3, DMN dorsal motor nucleus

Emphasis has been placed on the aggregation of endogenous α-synuclein in the gut as an initiating step in PD. However, if α-synuclein spreads from cell-to-cell in a prion-type fashion then the possibility of an exogenous source α-synuclein seeding aggregation is at least plausible. Correspondingly, it seems intuitive that dietary α-synuclein could seed aggregation in the gut. Active sampling of dietary α-synuclein or passive invasion via leaky gut may provide ample opportunity for the entry of misfolded α-synuclein. Exogenous α-synuclein, once it has entered the host tissue, could interact with several receptors important for the spread of α-synuclein pathology, namely LAG3, which are expressed on immune cells and/or neurons of the GI tract.^32,100–102^ Together, there is a potential prion source, mechanisms for uptake, and a possible explanation for the spread of α-synuclein in the gut (Fig. 2).

Dietary factors are only weakly associated with PD risk, and therefore, if dietary α-synuclein could seed aggregation, other factors (e.g., inflammation, aging and gut permeability) would likely be important co-mediators of the process. Indeed, many questions remain as to whether dietary α-synuclein could initiate PD pathology in the gut. However, it is difficult to ignore the fact that humans ingest α-synuclein whose receptors are expressed in cells responsible for antigen sampling in the gut and possibly in enteric neurons that are linked to the brain. The hypothesis could be directly tested by the oral administration of α-synuclein prion material to α-synuclein overexpressing mice, similarly to what has been done in studies of PrP^sc^.^242^ This study would be a necessary first step in addressing questions regarding the seeding capability of dietary α-synuclein.^242^
